# A prescription for health inequity: Building public health infrastructure in resource-poor settings

**DOI:** 10.7189/jogh.02.020302

**Published:** 2012-12

**Authors:** Asad Moten, Daniel F. Schafer, Elizabeth Montgomery

**Affiliations:** 1 Harvard University, Boston, Massachusetts, USA; 2 Institute for Translational Medicine and Novel Therapeutics, Healthnovations International, Houston, Texas, USA; 3 International Pediatrics AIDS Initiative, Houston, Texas, USA; 4 Washington State University, Pullman, Washington, USA; 5 Texas Children's Hospital, Houston, Texas, USA

 “My family has already purchased a coffin for me,” Naeema explained, enervated though she was. “The medicine we got from the local clinic has not helped. I am getting worse. We have spent almost all of our money going to regional hospitals to get a diagnostic test and request government subsidized medicine. My parents have sold everything we had – our crops, our land, and our livestock – to pay for my medicine. My family does not have any more money or even enough food to eat.” Naeema was HIV-positive, and due to a lack of diagnostic capabilities or availability of antiretroviral treatments (ARTs) in her village of Kibosho in Tanzania, she had been bed-bound for more than three months after the onset of her symptoms, when she first arrived at the clinic.

We live in a fortunate time: we have treatments and other essential tools we need to combat AIDS and other epidemics. Health is a human right. But interventions, either overpriced or merely nonexistent, are usually least available in places they are needed the most. A majority of deaths in these resource-poor settings are avoidable and demonstrate a failure of the health care infrastructure. In Kibosho, for example, even though the local rural clinician could test for HIV/AIDS antibodies in Naeema's blood, CD4 counts and antiretroviral treatment, which are the best options for improved quality of life and extended life expectancy, were simply not available to those living in the area. The result is non-action. It is not that there are no resources available to actually solve the problem, but rather a host of additional complications that prevent medications from reaching those who need them most, such as adequate roadways to rural communities, refrigeration units to preserve medications, and a host of others. Remedying these problems represent sustainable solutions to building public health infrastructures. In short, distributing medication is trivial if infrastructure to support distribution and administration of those medications is not prioritized. A paradigm shift, therefore, is needed to effectively address these issues. Such a shift would manifest itself in the form of established, local entities playing a central role not only in the distribution of medical aid but in wider systemic support needed to distribute medication and treat patients across resource-poor regions.

Sadly, Naeema’s story is not uncommon; tens of millions suffer due to inadequate care and lack of available resources. According to the UN Agency for HIV/AIDS [1] 27 million Africans live with the HIV or are dying of AIDS and some 35 million HIV-infected people live in the developing world, with women and children bearing a significant burden of disease. According to Rodriguez et al. [2], in 2005, when Naeema’s condition reached a critical point, only one in ten infected with HIV were tested and knew his or her HIV status. Since that time progress has been made in part due to PEPFAR, PMTCT, Global Fund and other programs, thus resulting in 20% knowing their status at present. Despite these far-reaching improvements, however, there is still much to be done. The fact that more than two decades into the epidemic the World Health Organization (WHO) reports only 80% of infected individuals are aware of their HIV status and 90% are unaware of their partner's status [3], is clear why the disease proliferates as it does and why it will be both exceptionally difficult to eradicate in the long run and challenging to contain the short term. According to the Joint United Nations Program on HIV/AIDS, of the 23 million Africans who are infected, fewer than 150 000 receive antiretroviral treatment [4]. Barring a miracle or a major paradigm shift by the international public health agents in both the public and the private sectors, these people, most of whom live in destitute communities, will die within the next decade. Despite the horrors of the pandemic, the international efforts to diagnose and subsequently deliver antiretroviral treatment to these marginalized populations are limited. It is a situation that we regard as a human rights calamity.

Perhaps the chief culprit for neglect of HIV/AIDS patients in the third world is heavy fragmentation of local health infrastructure in these countries. The term “global health infrastructure” refers to the conglomeration of public, private, and nonprofit efforts, whether unified or not, to address health issues. These entities, while promising to deliver tangible services and innovations, primarily succeed only in providing grandeur spectacles of delusional optimism and impractical idealism. This is exemplified in Naeema’s native Tanzania, where agents in both the public and the nonprofit sector have seemingly worked together to obtain ARTs for HIV/AIDS patients, but have largely failed to make them accessible to those who need them the most.

**Figure:**
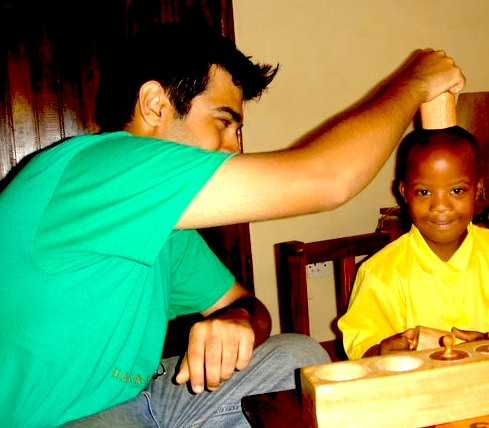
Photo: Courtesy of Aliasghar Saajan, Healthnovations International, Tanzania

Public and nonprofit agents have also failed to work toward developing a functional public health system in resource poor settings. In his 2009 article in *Philadelphia Social Innovations Journal* [5], David Hunter describes the current state of public health when he explains that “while nonprofits work incredibly hard, with passion and dedication, and often in incredibly difficult circumstances to solve society’s most intractable problems, there is virtually no credible evidence that most nonprofit organizations actually produce any social value.” He goes on to lament the fact that “Because so few nonprofits are willing to face this fact and ask themselves whether they are doing any good at all, ... we cannot rely on direct service nonprofits to fix themselves.” In other words, it can be argued that because nonprofit organizations don’t coordinate efforts internally or between other organizations, their work falls far short of its potential in working toward a viable solution.

The public sector is equally ineffective. Non-profit organizations bear the vast majority of the burden in caring for the world’s poorest because governments often refuse to take part in efforts to address issues like health infrastructure failure. Conventional wisdom in the global health beltway is that it is prohibitively expensive and logistically burdensome for governmental organizations to provide health care in resource-poor settings.

Government contribution is necessary in building health capacity in the any developing nation. Such involvement will serve to provide credibility and logistical support that cannot otherwise be orchestrated. Local legislative oversight will additionally ensure sustainability of with widespread disease, and also promote responsibility among those working in their own communities.

In conjunction with the failure of the public and private sectors to deliver better health outcomes, the epidemic of poverty is exacerbating health catastrophes across the developing world, including Naeema's native Tanzania. Consequently, a considerable portion of international development and humanitarian funding should be directed to building the capacity of the public health, public education and water sectors, all of which are essential to poverty alleviation and economic growth. In the case of the HIV/AIDS epidemic, even if ARTs were distributed to infected patients, such treatment would be rendered ineffective for many because they do not function properly for those that are malnourished. The WHO effectively describes the widespread positive impact a functioning public health infrastructure can have on communities in the developing world, while outlining the extensive social investment needed to secure such far-reaching, effective health service [6]:

“Strong, equitable and comprehensive health systems, which are designed to reach even the most marginalized communities, can help to mitigate some of those factors that entwine poverty, death and disease. Nevertheless, only by ensuring that all the functions of health systems (such as: service delivery; the health workforce; information; medical products, vaccines and technologies; financing; and leadership and governance) are driven by the guiding principles of social justice, social participation and inter-sectoral collaboration, will good quality healthcare that is accessible to all become a reality. “

As the WHO suggests, while health systems have a direct impact on health outcomes, they also work to alleviate economic hardship through job creation in local economies resulting from technology transfer, an emergent service industry based on mobilization and utilization of health care professionals, and a host of other task-based positions that will emerge as a direct result of a functioning infrastructure. Such an infrastructure, therefore, will serve to not only prevent the spread of disease itself, but effectively lessen the poverty-related hardship that can lead to disease faced in particular by rural communities in the third world.

Located in Southeastern Africa on the coast of the Indian Ocean, the per capita income for citizens of The United Republic of Tanzania is estimated to be at about US$ 524 a year, making it one of the poorest countries in the world, says United Nations Statistics Division [7]. It is also among the most affected countries in the sub Saharan region in terms of HIV/AIDS infection, with an estimated 1.5 million adults and children living with the disease. Because of the widespread and devastating effects of HIV/AIDS in the country, Tanzania serves as a useful model for the problems associated with treating disease and providing a framework for which potential solutions may be overlaid in a health care infrastructure.

Realizing the acute health needs of rural regions of Tanzania where health care infrastructure has been ravaged by rampant poverty and government neglect, it is conceivable that inequalities in access to quality medical care and the consequent health disparities in developing nations are perhaps the biggest challenges in public health today. If mass treatment of HIV/AIDS patients is to take place, ART is the most effective and likely means for it to take place. Circumstances are currently much more favourable in Tanzania than at the turn of the century, as ART has been available there for more than ten years. However, as estimated by the UNAIDS *World AIDS Day Report* [8,9], fewer than 20% of all the infected individuals, most of whom live in rural and hard to reach communities, are currently receiving treatment. This statistic bespeaks the fact that there is a desperate need for effective health infrastructure that can facilitate the delivery of needed care. Ongoing efforts in recent years, predominantly in the non-profit sector, have achieved substantial improvements in health access, prevention, and education [10]. But much more can and must be done. The general state of health systems throughout the developing world still severely limits the diagnosing and subsequent monitoring of HIV/AIDS and other patients in marginalized communities like Naeema’s.

Examining the history of both successful and failed attempts to wipe out HIV/AIDS and other infectious diseases provides considerable evidence that these diseases must be targeted jointly by both the public and the private sectors [11]. While public, non-governmental, and international health organizations will naturally focus on varying diseases at different times, eradication efforts have shown to be most effective when a significant number of groups in both private and public sectors coordinate their efforts and holistically tackle a particular disease with pragmatic solidarity. The public sector in this model of pragmatic partnership will initiate, fund, and ensure sustainability of equitable health care programs by building effective health systems in resource-poor settings while improving the skills and capacities of non-profit organizations. Additionally, the public sector will work to increase access of core competencies in the private sector to their most marginalized populations. Likewise, the private sector will expand the reach of public sector resources by targeting patients in their milieu. The WHO summarizes this strategy in noting that [6,12]:

“In many countries, the power of health interventions and technologies for curing disease and prolonging life is still not matched by the power of health systems to deliver these to people in need. It is essential to close this gap and the need is now strongly felt by the various actors in global public health. The desire to integrate the two mutually dependent dimensions – new resources for effective and affordable interventions and the broader fabric of health systems – into a more productive whole, that can deliver better health outcomes.”

Ultimately, it will be this idealistic yet pragmatic marriage of the public and private sectors, what the WHO calls “mutually dependent actors,” that will establish robust and effective health care infrastructures – a socially just paradigm that will deliver equitable health care to those like Naeema who need it the most in order to have a fair shot at a productive, healthy, and fulfilling life.
